# Ipsilateral Hemispheric Brain Atrophy in an Asymptomatic Child With Linear Morphea: A Case Report

**DOI:** 10.7759/cureus.21344

**Published:** 2022-01-17

**Authors:** Fahad Albadr, Hebah A Alnasser, Reem M Alshathri

**Affiliations:** 1 Radiology and Medical Imaging/Neuroradiology, King Saud Medical City/King Saud University, Riyadh, SAU; 2 Medicine and Surgery, King Saud University, Riyadh, SAU

**Keywords:** localized scleroderma, scleroderma, autoimmune, brain atrophy, linear morphea

## Abstract

Scleroderma is a family of systemic and local diseases that tighten and harden the skin and other connective tissues. Local scleroderma (i.e., morphea) typically involves the skin and underlying tissue causing progressive functional and cosmetic disturbances. While the etiology of scleroderma is unknown, it is correlated with autoimmune dysfunction. Linear morphea is a disorder that primarily affects children. This report describes the case of a nine-year-old girl with skin eruptions in the forehead, near the left eye, and in the anterior neck in addition to an underlying focal hemispheric frontal brain atrophy. There is no evidence of neurological deficits in this case. Linear morphea can lead to brain atrophy, causing several neurological dysfunctions such as seizures and cognitive impairment. Follow-up monitoring is critical, and also early recognition of new symptoms for optimal patient outcomes.

## Introduction

Scleroderma is a condition characterized by the tightening and hardening of the skin and other connective tissues, and it consists of several subtypes. Morphea is an autoimmune subtype of scleroderma that typically involves the skin with or without underlying tissues but elicits cosmetic and functional disturbances [1]. Morphea is divided into four subtypes: linear, generalized, plaque, and mixed [2]. A minority of morphea patients (20%) experience extracutaneous involvement, including arthritis, uveitis, and seizures [3]. Linear morphea is the most common subtype in pediatric patients and is more associated with neurological abnormalities than other forms of morphea [4]. We present a case of a young girl with skin eruptions in her face and neck in addition to an underlying focal hemispheric frontal brain atrophy but no apparent neurological deficits.

## Case presentation

A nine-year-old girl was clinically diagnosed with linear morphea at the age of seven, and she had a family history of autoimmune diseases in the form of hypothyroidism and hyperthyroidism. She first presented with skin eruptions over the forehead after trauma, and the lesions continued to spread over the neck and near her left eye within one year.

On physical examination, we noted a linear hypopigmented atrophic sclerotic plaque over the mid-forehead, two near the left eye, and one in the anterior aspect of her neck. We found no other skin or hair abnormalities. She was positive for proliferating cell nuclear antigen pattern on her antinuclear antibody testing workup. Given her history and physical exam findings, we diagnosed her with linear morphea.

**Figure 1:**
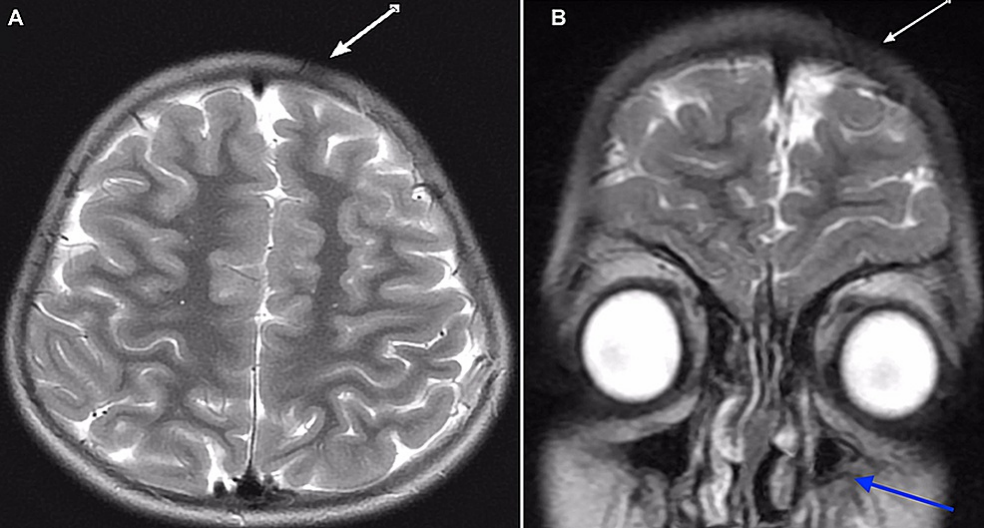
Sagittal and coronal T2-weighted magnetic resonance images demonstrate (A) a focal area of scalp thinning and subtle atrophy of the subcutaneous fat within the left frontal region without associated flattening or thinning of the underlying frontal bones and (B) no associated white matter abnormality. The left maxillary sinus is partially hypoplastic (blue arrow).

**Figure 2:**
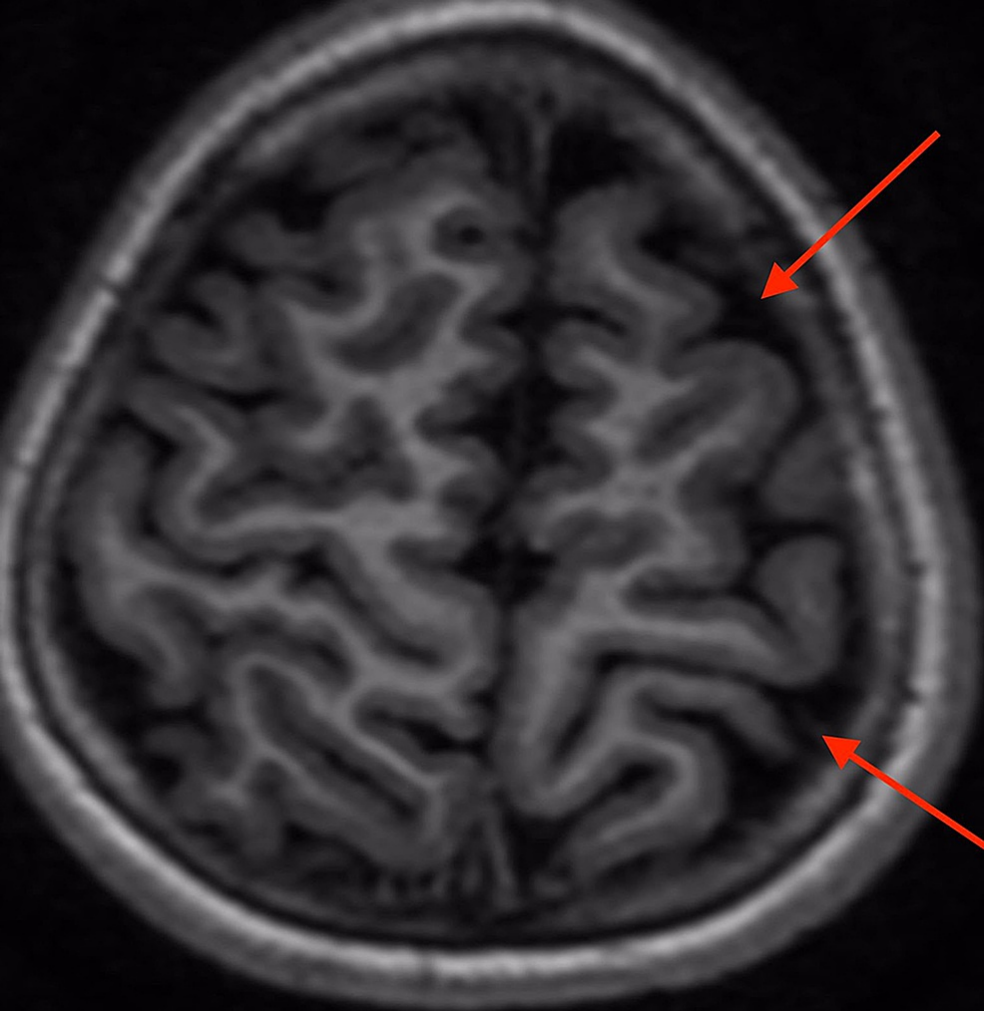
Axial T1-weighted magnetic resonance image showing subtle atrophy of the left cerebral hemisphere (red arrow). There is no cortical loss or abnormal gyral formation.

Her magnetic resonance imaging (MRI) of the brain revealed a focal area of scalp thinning and subtle atrophy of the subcutaneous fat (Figure 1A) and subtle focal atrophy of the left frontal subcutaneous fat with underlying mild atrophy of the left frontal lobe (Figure 1B). Figure 2 demonstrates another view of the brain MRI showing subtle atrophy of the left cerebral hemisphere. She is currently being treated with topical tacrolimus and topical steroids.

## Discussion

Linear morphea is an inflammatory skin disorder that can extend to the underlying tissue. Immune dysregulation, genetic predisposition, and environmental factors contribute to this disease, but the exact cause remains unknown [5]. Most patients (67%) are diagnosed before the age of 18 [1].

MRI may show intracranial changes, including calcifications and white matter-enhanced T2 lesions ipsilateral to the skin lesions, as well as focal brain atrophy. This has been demonstrated in both symptomatic and asymptomatic patients [6]. The most common lesions are intraparenchymal calcifications involving the basal ganglia, thalamus, and dentate nuclei [7]. Our patient had a localized left frontal lobe atrophy.

Our case is unique in terms of clinical presentation; she only experienced cosmetic changes with no neurological deficits such as seizures or cognitive impairment. To our knowledge, only a few cases of children have been reported with different levels of brain atrophy without evident neurological dysfunction [7,8]. A 2003 case described an 11-year-old boy with brain atrophy on the same side of the skin lesions and rapidly progressive symptoms, including seizures and cognitive dysfunction [9]. In 2004, a study in Montreal included nine patients monitored for three years. Five of these patients had no neurological symptoms despite having frontoparietal cortical changes on MRI. One of those five patients was a six-year-old girl [7].

The prognosis of linear morphea depends on the condition's severity, which is assessed using the Localized Scleroderma Skin Severity Index and the modified Rodnan skin score [1]. In a study in Italy with 133 patients, most participants achieved complete remission. Among those followed over 10 years, only 12.5% developed the active disease, all of which had the linear subtype [10].

A study was published in 2020 demonstrating the treatment approach for linear morphea, which is either medical or surgical depending on several factors, including the type, site, distribution, presence of extracutaneous involvement of the disease, feasibility, and cost. Medical treatment consists of local topical treatments such as steroids, imiquimod, and tacrolimus. Systemic treatment options include methotrexate-steroid combination, mycophenolate mofetil, hydroxychloroquine, cyclosporine, biologics, and phototherapy. A surgical approach is usually chosen for joint contractures or cosmetic correction, as seen in patients with en coup de sabre [3]. Delays in treatment are associated with higher relapse rates and more prolonged disease activity [10]. The most important diseases that can mimic linear morphea are acrodermatitis chronica atrophicans (i.e., Lyme disease) and linear melorheostosis. As opposed to linear morphea, linear atrophoderma does not present with inflammation or induration. Morphea presenting as hyperpigmented patches may be confused with fixed drug eruptions and macular lichen planus, which differ in their histopathological features. Morphea lesions can be misdiagnosed as acquired port-wine stains, delaying appropriate treatment [3].

## Conclusions

This case described a young girl with linear morphea who presented with skin eruptions in her face and neck with an underlying focal hemispheric frontal brain atrophy but no neurological deficits. Brain atrophy due to linear morphea is common and presents with or without clinical symptoms. This case highlights the importance of follow-up monitoring and early recognition of new symptoms for proper treatment.
